# Oncolytic viruses: a potential breakthrough immunotherapy for multiple myeloma patients

**DOI:** 10.3389/fimmu.2024.1483806

**Published:** 2024-10-30

**Authors:** Vincenzo Raimondi, Rosanna Vescovini, Mattia Dessena, Gaetano Donofrio, Paola Storti, Nicola Giuliani

**Affiliations:** ^1^ Laboratory of Hematology, Department of Medicine and Surgery, University of Parma, Parma, Italy; ^2^ Department of Medical-Veterinary Science, University of Parma, Parma, Italy; ^3^ Multiple Myeloma and Monoclonal Gammopathy Program, Department of Onco-Hematology, Azienda Ospedaliero-Universitaria di Parma, Parma, Italy; ^4^ Hematology Unit, Department of Onco-Hematology, Azienda Ospedaliero-Universitaria di Parma, Parma, Italy

**Keywords:** oncolytic viruses, multiple myeloma, immunotherapy, microenvironment, antitumor immunity

## Abstract

Oncolytic virotherapy represents an innovative and promising approach for the treatment of cancer, including multiple myeloma (MM), a currently incurable plasma cell (PC) neoplasm. Despite the advances that new therapies, particularly immunotherapy, have been made, relapses still occur in MM patients, highlighting the medical need for new treatment options. Oncolytic viruses (OVs) preferentially infect and destroy cancer cells, exerting a direct and/or indirect cytopathic effect, combined with a modulation of the tumor microenvironment leading to an activation of the immune system. Both naturally occurring and genetically modified viruses have demonstrated significant preclinical effects against MM cells. Currently, the OVs genetically modified measles virus strains, reovirus, and vesicular stomatitis virus are employed in clinical trials for MM. Nevertheless, significant challenges remain, including the efficiency of the virus delivery to the tumor, overcoming antiviral immune responses, and the specificity of the virus for MM cells. Different strategies are being explored to optimize OV therapy, including combining it with standard treatments and targeted therapies to enhance efficacy. This review will provide a comprehensive analysis of the mechanism of action of the different OVs, and preclinical and clinical evidence, focusing on the role of oncolytic virotherapy as a new possible immunotherapeutic approach also in combination with the current therapeutic armamentarium and underlying the future directions in the context of MM treatments.

## 1 Introduction

Multiple myeloma (MM) is a hematological malignancy that represents a significant therapeutic challenge due to its biological complexity, heterogeneity, and propensity to develop drug resistance (1, 2). Despite advances in therapeutic strategies, MM remains largely incurable, with frequent relapses and refractory disease (3).

In this context, the introduction of several immunotherapeutic approaches, such as monoclonal antibodies, bispecific antibodies, and CAR-T cell therapy, has changed the treatment landscape of MM. By harnessing the immune system’s ability to recognize and eliminate malignant cells, immunotherapy offers a promising way to overcome treatment resistance and improve patient outcomes (4–6).

Among the new emerging anti-cancer treatments, oncolytic virotherapy has gained considerable attention as a complementary and synergistic therapeutic modality within the MM treatment paradigm (7). Oncolytic virotherapy utilizes genetically engineered or naturally occurring viruses that selectively infect and replicate within neoplastic cells, leading to their lytic destruction while sparing normal tissues (8). This orchestrated cascade of events triggers stronger immunogenic responses, including activating innate and adaptive immune effectors against the tumor or reversing immunologically exhausted compartments (8, 9). Indeed, oncolytic viruses (OVs) exert profound immunomodulatory effects within the tumor microenvironment, reshaping the balance between pro-inflammatory and immunosuppressive signals (10). By targeting key immunoregulatory cell populations and promoting the recruitment and activation of cytotoxic lymphocytes, oncolytic virotherapy enhances the local and systemic antitumor immune response, thereby increasing therapeutic efficacy and durability (10); OVs also could sensitize refractory tumors to subsequent therapeutic interventions (11).

Nevertheless, despite the considerable therapeutic potential of oncolytic virotherapy, there are still significant challenges that require further investigation and clinical evaluations to demonstrate its efficacy and safety. These include optimizing viral delivery and dissemination strategies, enhancing tumor specificity and minimizing off-target effects, addressing immune evasion mechanisms related to pre-existing immunity, and developing combination approaches with novel immunotherapeutic strategies to enhance therapeutic synergy and overcome resistance to conventional treatments (12).

## 2 Oncolytic virus direct mechanisms and immunogenic effects in multiple myeloma

A well-known antitumor mechanism employed by OVs involves replicating within cancer cells and subsequently causing their direct lysis. This process is referred to as direct virus-induced oncolysis.

The aberrant molecular landscape and disrupted cellular homeostasis that characterize MM are closely related to the preferential targeting of OVs towards neoplastic plasma cells (PCs) (13).

OVs leverage the overexpression of specific surface receptors on cancer cells as a route of targeted cell entry. Several of these receptors, including membrane cofactor protein (CD46), junctional adhesion molecule-A (JAM-A), intercellular adhesion molecule-1 (ICAM-1), and decay-accelerating factor (DAF) are frequently upregulated in MM cells (14–17). These receptors serve as molecular gateways for OVs, facilitating viral attachment, entry, and subsequent oncolysis (14–17).

OVs also take advantage of other molecular alterations that are commonly found in MM cells. These alterations enable the replication of the viruses selectively, thus enhancing their efficacy.

A pivotal player in this context is the dysregulated RAS signaling pathway (13, 18). RAS-transformed cancer cells often exhibit defects in the innate antiviral defense mediated by the double-stranded RNA (dsRNA)-activated protein kinase (PKR) pathway. This compromised antiviral response makes cancer cells more susceptible to viral infection and facilitates unrestrained viral replication, leading to enhanced oncolysis (19–21). Similarly, OVs take advantage of the dysregulation of the phosphatidylinositol 3-kinase/Akt/mammalian target of rapamycin (PI3K/Akt/mTOR) axis. The activation of this axis enhances viral internalization and endosomal sorting, facilitating viral propagation within host cells (22, 23).

MM cells often exhibit defects in the interferon (IFN) pathway, which they exploit to evade immune surveillance, and consequently, OVs replicate in tumor tissues without interference from the antiviral effects of interferons (24, 25).

The therapeutic efficacy of OVs also depends on the indirect activation of the immune system against tumor cells.

**Figure 1:**
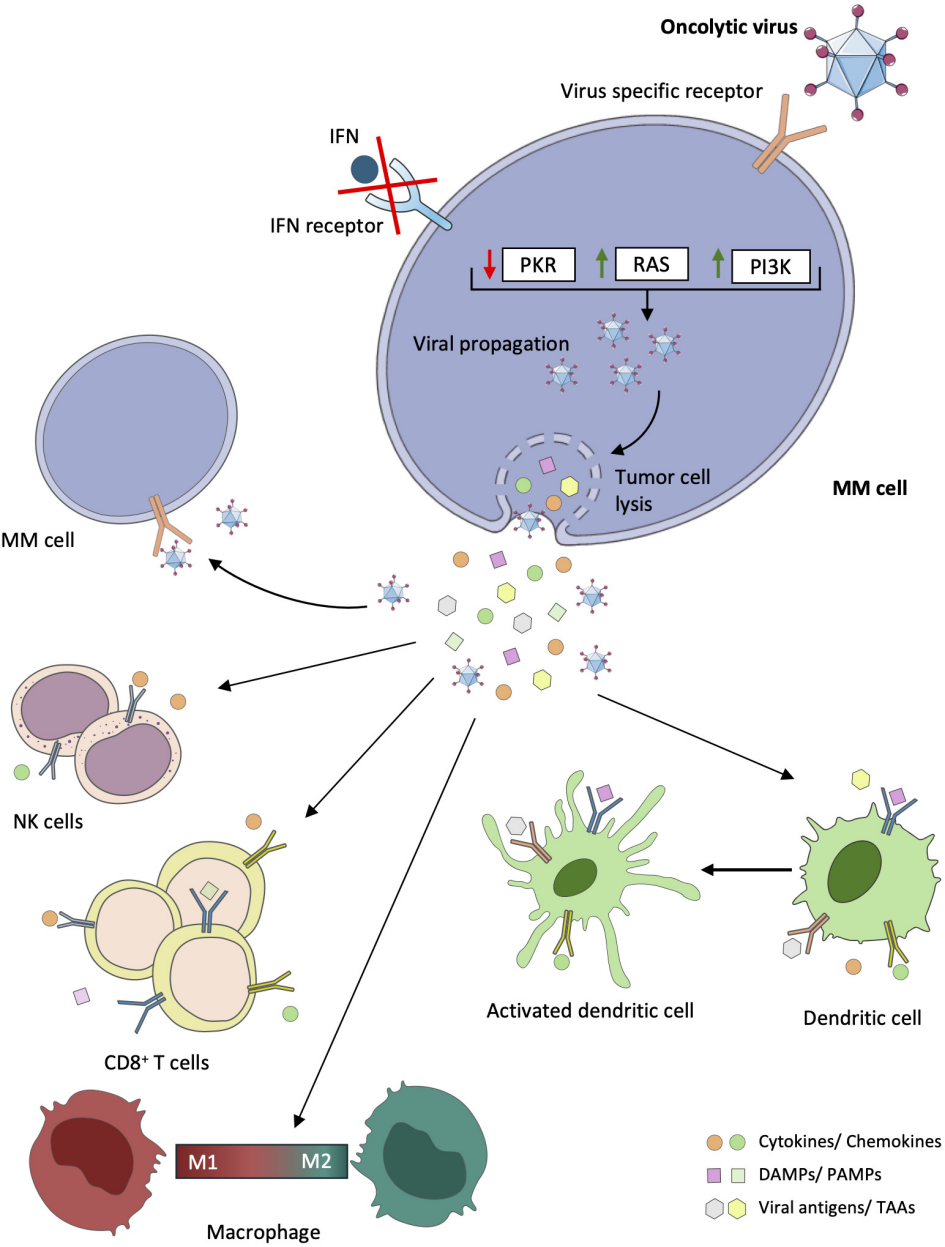
Direct and indirect antitumor effects of oncolytic viruses in multiple myeloma. In multiple myeloma (MM) cells, mutations or deletions are present in genes coding for key proteins of antiviral signaling pathways, including the interferon (IFN) pathway, the RAS pathway, the double-stranded RNA-activated protein kinase (PKR) pathway, and the phosphatidylinositol 3-kinase (PI3K) pathway. Consequently, oncolytic viruses (Ovs) can exploit these dysregulated signaling pathways in tumor cells to promote replication, infection, virus spread, and consequently lysis of tumor cells. Following viral infection, OVs can also induce tumor cell death through the mediation of immunogenic cells. Cytokines, viral elements (tumor-associated antigens (TAAs), viral pathogen-associated molecular patterns (PAMPs)), and cell damage-associated molecular patterns (DAMPs) can be released. These stimuli play a critical role in the recruitment and activation of immune cells, such as dendritic cells (DCs), natural killer (NK) cells, macrophages, and CD8^+^ T cells, which can reverse the immunosuppressive microenvironment that characterizes MM.

Indeed, following viral infection and tumor cell lysis, OVs induce immunogenic cell death (ICD) pathways within MM cells, triggering the release of cytokines, tumor-associated antigens (TAAs), and other danger signals, including damage-associated molecular pattern (DAMPs) and pathogen-associated molecular pattern (PAMPs). These are a potent stimulus for the maturation and activation of antigen-presenting cells (APCs), particularly dendritic cells, initiating a robust adaptive immune response against the tumor (24, 26–29). Additionally, virus-infected MM cells activate pattern recognition receptors (PRRs), such as Toll-like receptors (TLRs), thereby amplifying the immune response within the MM microenvironment (30). OVs also play a pivotal role in modulating the tumor microenvironment by polarizing infiltrating monocyte-derived macrophages from an M2 phenotype, associated with tumor-promoting activities, to an M1 phenotype, characterized by enhanced antitumor immune responses. This polarization is facilitated by the release of pro-inflammatory cytokines and chemokines from infected MM cells, reshaping the immune landscape to favor tumor suppression (28). Finally, some OV antigens or specific antigens loaded on genetically engineered OVs are implicated in reversing the exhausted T cell phenotype present in the tumor microenvironment. OVs can act as agnostic antigen vaccines, expanding the repertoire of cancer-specific neoantigens (29). **Figure 1** illustrates the various mechanisms through which OVs exert their anti-MM cell effects.

## 3 Oncolytic virotherapy for multiple myeloma: preclinical and clinical evidence

**Figure 2:**
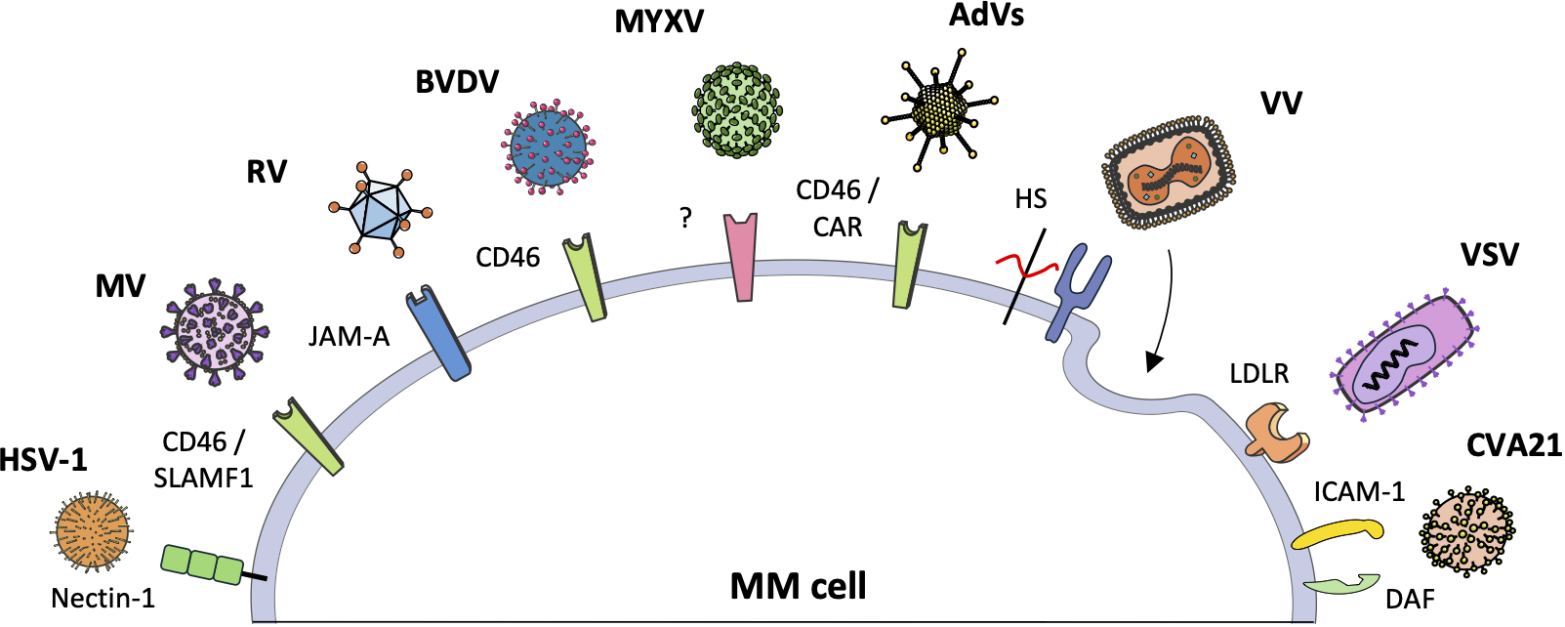
The interaction between oncolytic viruses and specific receptors in myeloma cells. Oncolytic viruses (OVs) can recognize and penetrate multiple myeloma (MM) cells through interaction with specific receptors on the cell surface. The figure illustrates the various OVs and the respective receptors with which they interact. Vaccinia virus and myxoma virus represent two distinct examples. Vaccinia virus employs endocytosis and membrane fusion to enter cells, forming syncytia. Myxoma virus has demonstrated efficacy against MM cells; however, its specific surface receptor remains unidentified.

Preclinical and clinical studies have examined the use of human and non-human viruses in the treatment of MM. Specifically, five RNA viruses (Measles virus, Reovirus, Coxsackievirus A21, Vesicular stomatitis virus, and Bovine viral diarrhea virus) and four DNA viruses (Adenovirus, Herpes simplex virus type 1, Vaccinia virus, and Myxoma virus) have been studied. These viruses have been evaluated in both monotherapy and combination therapy associated with chemotherapy and/or radiotherapy, as well as purging agents during autologous stem cell transplantation. Although these therapeutic viruses are derived from naturally occurring viral strains, they have been modified to increase their selectivity toward cancer cells or to improve their efficacy in eradicating MM. For instance, OVs have been engineered by integrating targeting ligands or peptides into their capsids to recognize MM-specific antigens or surface receptors, as well as utilizing tissue-specific promoters or enhancers to restrict viral gene expression within MM cells (24, 28, 31–34). Similarly, the integration of therapeutic transgenes into OVs facilitates targeted delivery of cytotoxic agents, immune modulators, or proapoptotic factors specifically to MM cells, thereby enhancing the oncolytic effect and potentiating antitumor immune responses within the MM microenvironment (23, 28, 35, 36). **Figure 2** illustrates the main receptors through which individual viruses enter MM cells, while **Table 1** lists the clinical studies conducted to date. The following sections will provide a detailed overview of all viruses studied for the treatment of MM, with an analysis of the underlying molecular mechanisms and preclinical and clinical results up to this point.

**Table 1:** List of oncolytic viruses currently being tested in myeloma clinical trials.

Virus	Name	Phase	Combination	Trial No.	Status	References
Measles (MV)	MV-NIS	I/II	± Cyclophosphamide	NCT00450814	Completed	(29, 44)
		II	Cyclophosphamide	NCT02192775	Completed	
		I	None	NCT03456908	Completed	
Reovirus (RV)	Reolysin	I	None	NCT01533194	Completed	(61)
		I	Lenalidomide or pomalidomide	NCT03015922	Unknown status	
		I	Bortezomib + dexamethasone	NCT02514382	Completed	(63, 64)
		I	Carfilzomib + dexamethasone	NCT02101944	Completed	(62)
		I/II	Bortezomib + pembrolizumab	NCT05514990	Recruiting	
		I	Dexamethasone + carfilzomib + nivolumab	NCT03605719	Completed	
Vesicular stomatitis virus (VSV)	VSV-IFNβ-NIS	I	± Cyclophosphamide	NCT03017820	Recruiting	(110)

### 3.1 Measles virus

Measles virus (MV) is a negative single-stranded RNA virus of the Paramyxovirus family. Its genome encodes six proteins, three of which are essential for viral envelope formation: matrix protein (M), hemagglutinin (H), and fusion protein (F) (37). The hemagglutinin facilitates entry into target cells by binding to cellular receptors such as CD46 and Signaling Lymphocytic Activation Molecule Family Member 1 (SLAMF1), which are particularly expressed in MM PCs, and Nectin-4 (14, 37). The vaccine strain currently used is the Edmonston strain (MV-Edm), which was first isolated from a patient in 1954. An important distinction among MV strains is their preference for specific host cell receptors: wild-type strains tend to prefer the SLAMF1 receptor, whereas it is known that MV-Edm selectively targets the CD46 receptor (37).

*In vitro* studies have shown excellent replication of MV-Edm in several MM cell lines and primary MM cells isolated from patients’ bone marrow (BM), and a limited replication in phytohaemagglutinin (PHA)-stimulated peripheral lymphocytes (38). MV-Edm infection induces significant cytopathic effects in MM cells, leading to the formation of multinucleated syncytia and subsequent cell death (38).

Moreover, MV-Edm demonstrated antitumor efficacy by inhibiting MM cell engraftment in xenografts in immunocompromised mice (38).

Recently, a correlation between CD46 receptor expression and the tumor suppressor gene Tumor Protein P53 (TP53) has been highlighted. Lok et al. showed that TP53-deficient MM cells exhibit increased CD46 expression, with an increased susceptibility to MV infection compared to cells with functional TP53 (39).

To enhance the oncolytic efficiency of the MV-Edm virus and facilitate non-invasive imaging of infected tissues, a recombinant version expressing human sodium iodide symporter (NIS) was developed (40). This modified virus, designated MV-NIS, exhibited a replication rate comparable to MV-Edm (40). MM cells infected with MV-NIS have been shown to efficiently incorporate radioiodine. Serial gamma chamber imaging showed the intratumorally spread of the virus and the uptake of iodine-123 (^123^I) in both MV-sensitive tumors that responded positively to MV-NIS treatment and non-responsive tumors. Notably, complete regression of MV-resistant tumors was observed when radioactive iodine, ^131^I, was administered 9 days after a single intravenous injection of MV-NIS (40).

To further optimize therapeutic efficacy, retargeted viruses derived from MV-Edm were developed. These viruses were engineered to selectively target MM cells, reducing side effects on healthy tissues. The incorporation of antibody fragments specific for PCs markers, such as CD38 or Wue-1, into the H envelope protein of the MV-Edm virus resulted in enhanced directional ability, contributing significantly to tumor growth inhibition and increased survival in animal models (31, 41).

In addition to direct strategies for MV treatment, alternative approaches have been developed involving the use of Cytokine-Induced Killer (CIK) cells as vectors for oncolytic therapy. CIK cells represent a heterogeneous subset of *ex vivo* expanded T lymphocytes that exhibit phenotypic and functional properties of both natural killer (NK) cells and T lymphocytes (42). The infected cells exhibited a high capacity to eradicate MM cells in both culture and animal models, significantly outperforming the efficacy of uninfected CIK cells (43).

In parallel, clinical trials, such as the Phase I/II trial NCT00450814, evaluated the efficacy of MV-NIS in the treatment of MM (29, 44). A total of 32 patients were enrolled in this study. The 29 evaluable patients had a median age of 62 years and a median of 5 prior therapies. In Phase I, 13 patients were initially enrolled to receive various doses of MV-NIS. Although some patients experienced adverse reactions, including severe neutropenia, the maximum tolerated dose was not reached, and TCID_50_ 10^11^ was established as the treatment dose for the Phase II trial (44). After confirming the safety of the initial doses, Phase 2 was designed with the addition of cyclophosphamide before MV-NIS treatment.

The study reported significant clinical improvement after MV-NIS treatment. One patient treated with TCID_50_ 10^11^ achieved a complete response lasting 9 months with an isolated relapse in the skull without recurrent BM involvement. Four other patients showed a transient reduction in serum immunoglobulin-free light chains of at least 25% during the first 4 weeks of therapy, indicating a possible response to therapy. One patient had a subjective reduction and shrinkage of his extramedullary plasmacytomas on his back and thighs (44).

A subsequent study based on the same trial suggests that MV-NIS boosts anti-MM T cell responses in MM patients (29). Before virotherapy, more than 50% of patients showed T cell responses against multiple tumor-associated antigens, indicating existing immune activity against cancer cells. After MV-NIS treatment, T lymphocyte responses against specific antigens such as MAGE-A3 and MAGE-C1 were significantly enhanced. Particularly encouraging was the case of a patient enrolled in the study with high levels of cytotoxic T lymphocytes reactive to MV and tumor antigens. This patient achieved long-term complete remission, highlighting the potential of MV-NIS in combination with other immunomodulatory agents to support durable tumor remission in MM patients (29).

### 3.2 Reovirus

Reovirus (RV), also known as respiratory enteric orphan virus, got its name because it was initially not associated with any known disease. The Dearing strain of reovirus, classified as serotype 3 and commercialized as Reolysin for therapeutic purposes, is a ubiquitous, non-enveloped human virus with a genome of 10 double-stranded RNA segments (45). RV employs the JAM-A as a means of entering tumor cells (46). MM cells exhibit high expression of JAM-A, particularly in advanced stages of the disease or in the presence of treatment resistance (15). The replication is facilitated by the MM cells’ suppression of the antiviral protein PKR, which allows for increased viral production (47). Altered PKR allows RV to circumvent this cellular defense mechanism, replicate efficiently, and cause lytic infection (30).

Several studies have demonstrated that RV induces endoplasmic reticulum (ER) stress and the expression of the pro-apoptotic protein NOXA, resulting in the apoptosis of MM cells (48, 49). RV also promotes autophagy in MM cells, contributing to reduced cell viability (50). In mouse models, the combination of RV with bortezomib has been shown to potentiate apoptotic activity, increasing ER stress and NOXA expression, while reducing MM tumor burden without significant adverse effects (48). Kennedy et al. identified nicotinamide adenine dinucleotide (NAD^+^) as a critical factor in the susceptibility of MM cells to reovirus-induced oncolysis. Pharmacological inhibition of nicotinamide phosphoribosyl transferase (NAMPT), a key enzyme in the NAD^+^ synthesis pathway, with FK866 sensitized MM cells to RV oncolysis, causing mitochondrial dysfunction and promoting autophagy and cell death (51). In addition, the concomitant administration of RV with histone deacetylase inhibitors (HDACi) resulted in an increased expression of JAM-A, rendering MM cells more susceptible to oncolytic action (52). Furthermore, RV demonstrated efficacy in the *ex vivo* purging of autologous stem cell transplantation, selectively killing MM cells while sparing healthy ones, thus improving therapeutic outcomes (53, 54).

Besides direct effects, it has been observed that RV exerts indirect effects on the immune system. In mouse models, RV has demonstrated the ability to significantly reduce tumor burden and MM-induced bone disease, which correlates with an increase in NK cells and effector memory CD8^+^ T cells (55). The combination of RV with lenalidomide or bortezomib has been shown to stimulate a robust antitumor immune response in preclinical studies (56–58).

The rationale for combining OV with bortezomib is based on the latter’s ability to also induce the ICD. This process is characterized by the exposure of calreticulin on dying MM cells, their phagocytosis by dendritic cells, and the induction of a specific immune response against MM (59).

The use of RV together with lenalidomide and dexamethasone has been demonstrated to overcome the resistance of MM cells to direct viral death by activation of NK cells (56). RV also reduces the protection offered by BM stromal cells, thereby improving the overall efficacy of the treatment by lenalidomide and dexamethasone (56).

Moreover, Thirukkumaran et al. observed that co-treatment with bortezomib reduces regulatory T cells (Tregs) and suppressive myeloid cells (MDSCs), thereby increasing the activity of immune effector cells (57). Specifically, the RV-bortezomib combination stimulates the production of pro-inflammatory cytokines such as interferon-gamma (IFN-γ), creating an inflammatory environment that potentiates the activity of NK and CD8^+^ T cells.

The increased expression of immune markers such as PD-1 and PD-L1 observed following RV treatment suggests that this approach could be particularly effective when combined with targeted therapies such as PD-1/PD-L1 inhibitors (60).

The clinical effects of RV in the treatment of MM patients have been studied in various clinical trials, demonstrating an acceptable safety profile but limited efficacy results (61–64).

In clinical trial NCT01533194, patients received two doses of Reolysin (3×10^9^ TCID_50_/day or 3×10^10^ TCID_50_/day) without experiencing dose-limiting toxicities (DLTs). Nevertheless, no significant objective responses were observed, with only a few patients achieving disease stability for up to eight months (61). Analyses indicated that viral resistance, limited antitumor immune response, and inadequate viral dosing may have reduced treatment efficacy. A critical factor that emerged was the lack of JAM-A receptor expression in the patient’s cells, which may have limited viral infection of MM cells, reducing treatment efficacy. Otherwise, RAS mutations, which are prevalent in patients with relapsed MM, did not demonstrate a significant correlation with treatment efficacy (61).

In a different trial (NCT02101944), the efficacy of a combined treatment regimen comprising Reolysin, carfilzomib, and dexamethasone was evaluated in MM patients who had demonstrated resistance to carfilzomib (62). Six patients completed 28-day cycles, during which reovirus infection was observed in the BM on day 9 of the first cycle. Two patients demonstrated partial responses; however, one of them developed a cytokine storm with severe symptoms, and the other one discontinued treatment due to fever and severe thrombocytopenia. This cytokine storm, the first observed in blood cancer patients treated with OVs, was associated with T-cell activation due to combination therapy. The observed clinical responses were attributed to the infection of MM cells, the recruitment of CD8^+^ and NK cells, the increased expression of activated PD-L1 and caspase-3, and the viral protein production in MM cells (61).

NCT02514382 trial evaluated the safety and efficacy of RV combined with bortezomib and dexamethasone in patients with relapsed and refractory MM who had previously undergone at least one course of therapy (63, 64). The combination was well tolerated, with most toxicities presenting as transient flu-like symptoms that could be managed with acetaminophen, antiemetics, or antidiarrheals. No DLTs were observed, indicating that the maximum tolerated dose was not reached. The 3×10^10^ TCID_50_ dose of Reolysin was administered for five consecutive days in 21- and 28-day treatment cycles. Six of eleven evaluable patients (55%) demonstrated a reduction in paraprotein levels. In patients who responded to the treatment, there was an association between viral proliferation and increased apoptosis, as indicated by the increase in cleaved caspase-3-positive cells. Immunohistochemical analysis revealed a significant increase in cytotoxic T cells in responders, suggesting that these cells cluster around MM cells. This spatial change in the tumor microenvironment could contribute to the efficacy of the treatment (64).

### 3.3 Adenovirus

Adenoviruses (AdVs) are non-enveloped, double-stranded DNA viruses with an icosahedral capsid primarily composed of hexon, penton, and fiber proteins, belonging to the Adenoviridae family. In humans, over 100 AdV types have been identified, and classified into seven genetically distinct species (A–G) based on phylogenetic analysis of their genomic sequences, pathobiology, and immunological and tumorigenic properties (65). Although human AdVs cause significant numbers of respiratory, ocular, and gastrointestinal diseases, severe AdV-associated illness occurs predominantly in immunocompromised individuals. In the general population, AdV infections are typically self-limiting and lead to lifelong immunity (66).

Among AdV types, Ad5 (species C) is the most widely used vector for oncolytic virotherapy, having demonstrated success in both preclinical and clinical trials across various cancers (67). The infection of tumor cells by Ad5 begins with viral fiber knob attachment to receptors on the surface of malignant cells. The receptor specificity differs according to the viral serotype. For instance, Ad5 binds preferentially to the Coxsackie and Adenovirus Receptor (CAR), while Ad3 binds to desmoglein-2, CD46, or CD80/86 (68). This receptor diversity is particularly relevant to MM, where CD46 and CAR are variably expressed on malignant cells (69).

Preclinical studies have demonstrated the therapeutic potential of AdVs in MM. In one investigation, AdVs were employed as a therapeutic tool for purging MM cells, showing their ability to deliver the TK gene into MM cells using the DF3/MUC1 tumor promoter, with tumor cell transduction observed to be highly efficient (>80%) (34). Treatment with ganciclovir selectively eliminated MM cells without affecting normal progenitor cells (34). Senac et al. demonstrated that distinct AdV serotypes, including Ad5, Ad6, Ad26, and Ad48, can effectively infect and destroy MM cells while exhibiting minimal cytotoxicity against CD138^-^ cells. Ad5 and Ad6 exhibited a high capacity to infect MM cells through CAR and integrin receptors, while Ad26 and Ad48 utilized alternative receptors, such as CD46 and sialic acid (69).

Innovative strategies have been developed to enhance the therapeutic efficacy of Ad5 in MM treatment, taking advantage of its amenability to genetic modification. Through targeted genetic engineering, Ad5 can be optimized to improve tumor specificity and enhance its oncolytic efficiency, thereby increasing selectivity for malignant cells while reducing off-target cytotoxicity. Fernandes et al. developed an oncolytic AdV, AdEHCD40L, which expresses CD40 ligand (CD40L) under the control of hypoxia-specific promoters (32). This vector has been demonstrated to effectively inhibit the growth of MM cell lines *in vitro* and to significantly reduce tumor volume in mouse models. The therapeutic efficacy of AdEHCD40L has been attributed to both direct viral lysis and CD40L-mediated induction of apoptosis, suggesting a dual mechanism of action (32). Wenthe et al. investigated the use of AdVs LOAd700 and LOAd703, which had been modified to express trimerized CD40 ligand (CD40L) and 4-1BB ligand (4-1BBL), respectively (28). The viruses demonstrated potent oncolytic activity against MM cell lines and the activation of antitumor immune responses. LOAd703 demonstrated superior efficacy in controlling tumor growth in xenograft models, as evidenced by its ability to stimulate cytotoxic T cells and increase the expression of death receptors such as Fas (28).

Further therapeutic approaches have also demonstrated significant potential. Tong et al. combined an oncolytic AdV expressing TRAIL (ZD55-TRAIL) with the PI3K inhibitor LY294002 (23). This combination enhanced the cytotoxicity of the virus toward MM cells by inhibiting the Akt/mTOR survival pathway and enhancing the induction of apoptosis in tumor cells. Furthermore, the addition of the proteasome inhibitor MG132 resulted in a further increase in the expression of Death Receptor 5 (DR5), thereby sensitizing MM cells to ZD55-TRAIL-induced apoptosis (23).

Stewart et al. developed an oncolytic AdV, ADCE1A, which employs the MM-specific CS1 promoter to regulate E1A gene expression. This AdV demonstrated selective infection and replication in MM cell lines and induced oncolysis in CD138^+^ cells of MM patients, without affecting non-tumor cells (70).

Moreover, the combined use of a recombinant p53 AdV (rAd-p53) and bortezomib showed synergistic inhibition of proliferation and induction of apoptosis in MM cells. rAd-p53 enhanced the expression of p21, arresting the cell cycle and reducing the expression of cyclin B1, thus improving the efficacy of bortezomib treatment (71).

However, there are significant limitations to the use of Ad5 as a vector. First, an estimated 50–90% of the adult population is seropositive for pre-existing anti-Ad5 neutralizing antibodies (72, 73). These neutralizing antibodies have been shown to limit the antitumor efficacy of Ad5, particularly during systemic intravenous delivery (74–76). Second, intravenous administration of Ad5 can lead to liver toxicity due to significant liver sequestration, driven by Ad5 hexon binding to coagulation factor X (FX) (77, 78). In response to this, Alba et al. developed FX-binding-ablated Ad5 hexon vectors to mitigate this side effect (79).

To circumvent these limitations, researchers have begun exploring alternative AdV serotypes. For example, the low seroprevalence of antibodies against species D AdVs, coupled with their lack of FX binding, makes them promising candidates for further exploration as oncolytic agents (80). Additionally, chimeric AdVs have been developed to evade neutralizing antibodies (81).

### 3.4 Herpes simplex virus type 1

Herpes simplex virus type 1 (HSV-1) is a double-stranded DNA virus with icosahedral symmetry, belonging to the family Herpesviridae. It is primarily known to cause oral infections such as cold sores (82). Recently, oncolytic versions of HSV-1 (oHSV-1) have shown a promising ability to selectively infect tumor cells (83). This specificity is defined by the surface glycoproteins of individual virions, which interact with cell surface receptors, particularly Nectin-1 and Herpes Virus Entry Mediator (HVEM) (82). These receptors are highly expressed in MM cells, contributing to the selectivity of oHSV-1 infection (84).

Ghose et al. demonstrated that oHSV-1 effectively infected MM cells *in vitro*, causing apoptosis through cleavage of caspase-3. In murine models, infection with oHSV-1 led to a significant reduction in tumor volume (84). Furthermore, the combination of oHSV-1 with NK cells immunotherapy has been demonstrated to enhance therapeutic efficacy through the activation of NK cells and the subsequent increased release of cytokines and cytotoxic capacity (85).

Additional therapeutic combinations including oHSV-1 with bortezomib or lenalidomide have shown synergistic effects (25, 86). Specifically *in vitro*, HSV1716 (SEPREHVIR^®^), when combined with bortezomib, prevented MM cell regrowth for up to 25 days (86); at the same time, third-generation HSV-1 T-01, when used together with lenalidomide, increased the cytotoxic effect and enhanced antitumor activity through the activation of NK cells and the modulation of the immune environment (25).

### 3.5 Coxsackievirus A21

Coxsackievirus A21 (CVA21) is a member of the Picornaviridae family and is a non-enveloped virus with an icosahedral structure and a genome consisting of a single strand of positive-sense RNA. In humans, natural infections of CVA21 are generally asymptomatic and not associated with severe disease (87). ICAM-1 and/or DAF cell surface receptors are responsible for the specific adhesion of CVA21 and subsequent infection of the host cell (88). CVA21 can bind to DAF expressed on the cell membrane but is unable to infect a cell unless ICAM-1 is co-expressed on the cell surface. Consequently, ICAM-1 is considered a pivotal factor in CVA21 entry, uncoating, and replication under normal infection conditions (88). In comparison to most non-malignant cells, MM cells express ICAM-1 and DAF at relatively high levels, which allows for selective oncolysis by CVA21 (16). The elevated level of ICAM-1 expression in MM cells can be attributed to the constitutive activation of the transcription factor NF-κB, which is present in numerous cell lines and patient biopsies (89).

*In vitro* studies have revealed that MM cell lines incubated with different concentrations of CVA21 exhibit a rapid cytopathic effect, even at low doses (16, 90). The oncolytic ability of CVA21 was confirmed in MM patients’ BM mononuclear cells, demonstrating that this effect is not limited to laboratory-adapted MM cell lines but is also effective in primary tumor samples infected *ex vivo*. Following infection with high levels of CVA21, a substantial clearance of tumor cells was observed, with minimal effects on non-malignant cells (16).

In SCID mice with human MM xenografts, a study demonstrated that the tumors exhibited rapid and complete responses to treatment with CVA21, either by intravenous or intratumorally administration (90). However, once the tumors regressed, the mice developed hind-limb paralysis and died rapidly. Pathological analysis revealed the complete ablation of tumor tissue, accompanied by the presence of diffuse myositis in the muscle tissues. CVA21 virus was recovered from muscle biopsies, but no evidence of central nervous system infection was found. Toxicity was observed in tumor-bearing animals with a dose of CVA21 up to 560 TCID_50_. To mitigate myositis, adenoviral vectors encoding for mouse IFN-α were administered before CVA21 therapy. However, the impact on tumor response or survival was minimal (16). Ongoing studies aim to find potential alternatives to reduce off-target effects.

### 3.6 Vaccinia virus

Vaccinia virus (VV) is a double-stranded DNA virus belonging to the Poxviridae family, best known for its use in the eradication of smallpox in the 1970s (91). This virus employs several mechanisms to enter host cells. Once attached to the cell surface via specific receptors, such as heparan sulfates, VV exploits the process of endocytosis mediated by macropinocytosis to penetrate the cytoplasm (91). Although the mechanism of high selectivity of VV for MM cells is not fully elucidated, it is postulated that aberrant signaling pathways through the RAS/Mitogen-Activated Protein Kinase (MAPK) pathways may contribute to this effect (91).

Attenuated VV variants with specific genetic deletions have been developed to selectively infect and replicate in malignant PCs, minimizing toxicity in normal tissues. A modified VV with double genetic deletion and insertion of a reporter gene has been shown to effectively infect several MM cell lines, inducing apoptosis and reducing cell viability *in vitro*, as well as slowing tumor growth and improving survival in MM mouse models without causing significant damage to healthy tissues (92). Another approach involved the use of a VV regulated by let-7a microRNA and with a deletion of the thymidine kinase gene (ΔTK), which demonstrated preferential localization of the virus in MM cells and reduced systemic toxicity. This engineered virus showed significant antitumor effects and improved survival in SCID mice (93). A further study explored the use of two novel VVs (TK-deletions) as vectors for anti-cancer gene delivery, miR-34a and Smac, respectively (36). The results demonstrated that the novel oncolytic VVs can effectively infect MM cell lines and significantly enhance exogenous gene expression. Furthermore, the combined use of VV-miR-34a and VV-Smac exhibited a synergistic effect by inhibiting tumor growth and inducing apoptosis *in vitro* and *in vivo*. The proposed underlying mechanism is that blockade of Bcl-2 by VV-miR-34a increases cytochrome c release from mitochondria and thus synergistically amplifies the antitumor effects of Smac-induced cell apoptosis (36). Finally, a VV modified to express Beclin-1 protein demonstrated the ability to induce significant autophagic cell death in MM cells by activating Sirtuin 1 (SIRT1) protein and promoting deacetylation and transfer of Microtubule-Associated Protein 1A/1B-light chain 3 (LC3) from the nucleus to the cytoplasm. This suggests a novel mechanism of action that could overcome the resistance of cancer cells to apoptosis (35).

Preclinical data regarding the use of VV in the treatment of MM find confirmation in a significant clinical case involving a 67-year-old patient with IgA-type MM (94). The patient received intravenous injections of a specific variant of VV, known as the AS strain, which resulted in a substantial reduction in monoclonal IgA levels and an increase in NK cell activity. It is noteworthy that no significant adverse effects were observed throughout treatment, which suggests a favorable safety profile for VV (94). This clinical case not only demonstrates the efficacy of VV in reducing disease biomarkers but also its potential to improve the patient’s immune response.

### 3.7 Myxoma virus

Myxoma virus (MYXV) belongs to the genus Leporipoxvirus of the family Poxviridae. It possesses a large linear double-stranded DNA genome enclosed in a brick-shaped virion (95). The entire MYXV replication cycle takes place in the cytoplasm of infected cells, where the virus produces a variety of immunomodulatory proteins that interact with the host. In the wild, MYXV exclusively infects rabbits and European brown hares and is not pathogenic to other hosts (95). Nevertheless, MYXV is capable of replicating in human tumor cell cultures, which exhibit a particular degree of permissiveness towards this virus. This permissiveness is attributed to its interaction with deregulated cellular pathways, particularly the Akt pathway (96). MM cells exhibit alterations in this pathway, rendering MYXV a potential oncolytic agent for the treatment of this tumor type (13).

The mechanism of action of MYXV differs from traditional oncolytic approaches, as it induces apoptosis in MM cells through the activation of caspase-8. This process is due to the depletion of apoptosis inhibitory proteins (cIAPs) caused by virus-mediated translational arrest (97). Additionally, MYXV induces autophagy, as evidenced by increased expression of the key proteins ATG-5, Beclin-1, and LC3B, and the presence of autophagosomes (98). Furthermore, the virus is capable of inhibiting the Activating Transcription Factor 4 (ATF4) expression and reducing the Myeloid Cell Leukemia 1 (Mcl1) levels, thereby overcoming resistance to proteasome inhibitors (99). A recent study investigated the combination of MYXV with lenalidomide and bortezomib, revealing a significant reduction in MM cell viability and an increase in early apoptosis. This synergistic effect is mediated by increased caspase-9 expression (100). In mouse models, the systemic administration of MYXV demonstrated efficacy in eliminating MM cells by inducing robust CD8^+^ T antitumor immune responses, suggesting the possibility of its use as a systemic therapy in patients (101).

De Matos et al. investigated the therapeutic potential of armed MYXV, engineered to express immunomodulatory proteins such as IL-12 and decorin. The authors reported significant oncolytic effects and transgene expression in MM cell lines, suggesting that this new approach could be applied in patients resistant to other immunotherapy strategies (102).

As with other previously treated viruses, MYXV has been demonstrated to be effective in purging *ex vivo* autologous hematopoietic stem cells (HSPCs) contaminated with MM cells, while preserving normal HSPCs and reducing post-transplant recurrence (103). Furthermore, *ex vivo* virotherapy with MYXV demonstrated encouraging outcomes in optimizing allogeneic hematopoietic cell transplantation. This approach significantly reduced the proliferation of alloreactive T cells and prevented graft-versus-host disease (GVHD) without compromising the antitumor effect (27). Finally, infusion of autologous leukocytes preloaded *ex vivo* with MYXV revealed remarkable efficacy in targeting minimal residual disease (MRD) of MM, suggesting a promising approach to overcome drug resistance and improve survival rates (104).

### 3.8 Vesicular stomatitis virus

Vesicular stomatitis virus (VSV) is an enveloped, single-stranded, negative-sense RNA virus belonging to the Rhabdoviridae family (105). It employs surface molecules such as the low-density lipoprotein receptor (LDLR) to target cells. LDLRs, due to their ubiquitous expression, enable VSV to infect a diverse range of cell types. However, VSV infection is typically inhibited by the activation of PKR and the production of IFN. Given that the PKR system is defective in cancer cells, VSV exhibits high selectivity (106).

In MM, preclinical studies have demonstrated promising results for the use of VSV both *in vitro* and *in vivo* (107). An attenuated variant, VSV(Δ51)-NIS, with a deletion of methionine 51 in the matrix protein and expression of the NIS gene, demonstrated specific oncolytic activity against MM cell lines and primary MM cells, without causing neurotoxicity in mouse models (108). Infection was monitored noninvasively by serial gamma camera imaging of radioactive iodine biodistribution. The combination of VSV(Δ51)-NIS with ^131^I further enhanced the efficacy of tumor regression and survival in immunocompetent mice (108). In addition, another study demonstrated that a VSV-IFNβ construct, which expresses IFN-β, significantly prolonged the survival of mice with disseminated MM (24). This was achieved through the combined action of the oncolytic activity of VSV and the immunomodulatory properties of IFN-β. VSV-IFNβ demonstrated specific oncolytic activity against human MM cells and primary patient samples, although with variable susceptibility (24). Moreover, the combination of VSV with bortezomib demonstrated synergistic effects *in vivo*, despite the antagonism observed *in vitro*. This suggests that the enhanced therapeutic efficacy observed may be mediated by host immune responses (109).

These promising results led to the design of a phase I clinical trial (NCT03017820) to assess the safety and optimal dosing of VSV-IFNβ-NIS in patients with relapsed or refractory MM. The study included 15 patients with relapsed/refractory hematologic malignancies, of whom 7 had MM (110). Patients received a single intravenous infusion of VSV-IFNβ-NIS across four dose levels (DL), with the highest dose being 1.7 × 10¹¹ TCID_50_ (DL4). No dose-limiting toxicities were observed, although 1 of 2 MM patients treated at DL4 experienced grade 2 cytokine release syndrome, which was transient and resolved within 24 to 48 hours (110). Overall, MM patients did not show significant clinical responses, with disease stabilization being the best outcome observed. Notably, one MM patient had an osteolytic lesion in the right ilium that showed increased ^99m^Tc-pertechnetate uptake on days 1 and 5 post-treatment, indicating viral presence. This lesion also demonstrated reduced [^18^F]Fluorodeoxyglucose activity on PET/CT, suggesting an initial response to therapy, although the patient experienced overall disease progression. A separate lesion in the left acetabulum showed no changes post-treatment, with increased size and bone destruction observed at the 6-month follow-up (110). Additional study arms have been added to this ongoing phase I trial to explore the safety and efficacy of combining VSV-IFNβ-NIS with drugs that modulate antiviral or antitumor immune responses.

### 3.9 Bovine viral diarrhea virus

Bovine viral diarrhea virus (BVDV) is a small, enveloped RNA virus belonging to the genus Pestivirus of the family Flaviviridae (111). BVDV is a significant pathogen of cattle, causing syndromes affecting the intestinal, respiratory, and reproductive systems. It is important to note that BVDV is not pathogenic to humans (111). It has been demonstrated that BVDV utilizes CD46 as a receptor for entry into host cells, a process analogous to that observed with the MV (112). Furthermore, several studies have shown that members of the heparan sulfate family, including the CD138 molecule (a distinctive marker of MM), act as cellular receptors for BVDV binding to host cells (113).

Marchica et al. investigated the efficacy of BVDV in the treatment of MM cells, emphasizing its potential as a novel therapeutic strategy (17). Specifically, BVDV demonstrated a selective cytotoxic effect on MM cells, with a significant increase in cell death and activation of apoptotic markers. In *ex vivo* experiments, BVDV treatment significantly reduced the percentage of viable CD138^+^ cells within mononuclear cells isolated from BM aspirates of MM patients, without altering the viability of other cell populations. Moreover, pretreatment with bortezomib markedly augmented the cytotoxic impact of BVDV on MM cell lines, indicating a synergistic effect resulting from the activation of the caspase-3-mediated apoptotic pathway (17).

Finally, in mice injected subcutaneously with MM cells, BVDV treatment significantly reduced tumor burden without evidence of toxicity to vital organs such as the heart and lungs (17).

## 4 Challenges and optimization strategies in myeloma oncolytic virotherapy

Oncolytic virotherapy presents a promising avenue for treating MM, but it faces significant challenges related to delivery, immune evasion, and biosafety that must be meticulously addressed (114).

In diseases such as MM, characterized by systemic involvement, intravenous (i.v.) delivery of OVs provides a feasible strategy to target malignant cells residing in the BM and other extramedullary sites (114). However, systemic delivery introduces several obstacles, including poor extravasation from tumor vasculature, non-specific sequestration in the liver, and rapid neutralization by pre-existing antiviral antibodies or treatment-induced neutralizing anti-viral antibodies. Neutralization by the immune system, especially in immunocompetent hosts, limits the therapeutic potential of OVs by reducing their circulation time and preventing effective tumor targeting (115). Various strategies have been developed to circumvent these issues. For instance, PEGylation, a polymer-based technique, has been shown to shield OVs from antibody-mediated neutralization and prevent nonspecific accumulation in the liver, thereby prolonging their systemic half-life (116). Similarly, encapsulation in nanoparticles, including graphene, has demonstrated efficacy in protecting viral particles during systemic circulation (117). In addition, “stealth” viruses, such as MeV-Stealth, have been engineered to evade pre-existing immunity (118). MeV-Stealth, specifically re-engineered to evade neutralizing antibodies in measles-immune patients, has demonstrated promising results in targeting CD46-expressing MM cells (118). An equally significant challenge is the rapid clearance and liver trapping observed particularly with AdV (119). Ad5, as described above, binds to coagulation FX, which mediates liver transduction, leading to off-target effects and reduced therapeutic efficacy (79). To mitigate this, researchers have genetically modified Ad5 to remove its FX-binding domain, preventing sequestration in the liver without compromising its oncolytic function (79). Moreover, CAR-independent infection mechanisms have been developed to enhance tumor-specific targeting while avoiding liver trapping, a critical advancement for systemic virotherapy (120).

To further improve the efficacy of OVs, immunosuppressive agents such as cyclophosphamide have been co-administered to transiently suppress both innate and adaptive antiviral responses. Cyclophosphamide not only enhances viral replication but also depletes regulatory T cells, rebooting the immune system and promoting an enhanced antitumor response (121). Ruxolitinib, a JAK/STAT inhibitor, has also been employed to enhance viral replication by inhibiting interferon responses in IFN-competent tumor cells, allowing for improved OV propagation while mitigating the risk of excessive cytokine release or rapid tumor lysis syndrome (122). In addition to these pharmacological interventions, the use of non-human or rare viruses presents a promising alternative to circumvent the issue of pre-existing immunity. These viruses, being non-pathogenic to humans, are unlikely to encounter neutralizing antibodies in the human population (123). However, they do pose biosafety risks, including potential shedding or recombination with wild-type viruses, underscoring the need for stringent safety protocols in clinical settings (114). While clinical trials involving OVs such as MV-NIS, Reolysin, and VSV-IFNβ-NIS in MM have shown acceptable safety profiles, with responses ranging from partial remission to disease stabilization, the risk of latent viral infections or recombination remains a concern (29, 44, 61–64, 110). For instance, HSV-1, which serves as the backbone for T-VEC, the first FDA-approved oncolytic virus, has been associated with latent infections, highlighting the importance of long-term safety surveillance (124). In the case of the MV-NIS trial specifically, viral shedding in sputum and urine was typically limited to the first 8–15 days following therapy, suggesting a controlled window of viral presence post-treatment (44). Nonetheless, the potential for reactivation or recombination, as seen with other viral platforms, warrants close surveillance even after the acute phase has passed.

A critical advantage of OVs is their potential to synergize with immune-based therapies, such as bispecific T-cell engagers (BiTEs) and CAR-T cell therapies (125, 126). OVs can serve as genetic engineering platforms to express BiTEs, which can redirect T cells to target tumor-specific antigens without relying on MHC-I antigen presentation (125). However, due to their small molecular weight and short half-life, BiTEs typically require continuous infusion, which increases the risk of systemic adverse effects (127). The selective replication of OVs in tumor cells provides a means to restrict BiTE expression to the tumor microenvironment, reducing off-target effects and enhancing therapeutic specificity (125). Moreover, OVs can reshape the tumor microenvironment by inducing the production of pro-inflammatory cytokines and chemokines, which promote the infiltration and activation of CAR-T cells (126). This ability to reprogram the immunosuppressive tumor niche is particularly valuable in MM, where the tumor microenvironment often limits the efficacy of CAR-T cell therapy alone (126).

Genetic modifications of OVs to enhance tumor tropism are also crucial. The heterogeneity of MM, along with the variability in viral receptor expression across patients, complicates the implementation of uniform treatments. Investigating differentially expressed genes in MM cells and designing ligand-pseudotyped OVs that target these specific markers could significantly enhance tumor selectivity (11).

Overall, continued research is needed to optimize safety and efficacy of OVs in MM, including monitoring for long-term risks such as viral persistence or recombination. Nevertheless, the integration of OVs with immunotherapies holds significant promise for overcoming the current barriers in MM treatment.

## 5 Conclusions

Over the past decade, in the field of oncolytic virotherapy, significant progress has been made, in exploring the use of different viruses, both natural, engineered, and of non-human origin. This innovative approach is particularly promising in the context of immunotherapy for the possible capacity to potentiate the effect of other drugs and to potentiate immune-mediated MM cell death. Oncolytic virotherapy may also represent an innovative strategy for a possible personalized approach due to the specific characteristics of tumoral cells and their microenvironment that make them susceptible to viral therapy. While OVs show potent anti-MM activity both *in vitro* and *in vivo* in pre-clinical mouse models, their clinical use as monotherapy has limitations. To maximize therapeutic efficacy, it is critical to combine OVs with other therapeutic agents including proteasome inhibitors and immunotherapeutic agents such as immunomodulatory imide drugs, monoclonal antibodies, and T cell therapy.

In conclusion, the future success of oncolytic virotherapy in the treatment of MM will depend on key factors such as the identification of biomarkers predictive of tumor response, the improvement of administration strategies, and the enhancement of the immune response through combination approaches. It is hopeful that through continued research and the integration of innovative therapeutic strategies the full potential of oncolytic virotherapy can be realized with an appropriate clinical development for MM patients.
